# Mechanisms of Allergen Immunotherapy and Potential Biomarkers for Clinical Evaluation

**DOI:** 10.3390/jpm13050845

**Published:** 2023-05-17

**Authors:** Umit M. Sahiner, Mattia Giovannini, Maria M. Escribese, Giovanni Paoletti, Enrico Heffler, Montserrat Alvaro Lozano, Domingo Barber, Giorgio Walter Canonica, Oliver Pfaar

**Affiliations:** 1 Pediatric Allergy Unit, Department of Pediatrics, Hacettepe University School of Medicine, Hacettepe University Childrens Hospital, 06230 Ankara, Turkey; 2 Allergy Unit, Meyer Children’s Hospital IRCCS, 50139 Florence, Italy; 3 Department of Health Sciences, University of Florence, 50139 Florence, Italy; 4 Departamento de Ciencias Médicas Básicas, Instituto de Medicina Molecular Aplicada (IMMA) Nemesio Díez, Facultad de Medicina, Universidad San PabloCEU, CEU Universities, 28668 Madrid, Spain; 5 Department of Biomedical Sciences, Humanitas University, Pieve Emanuele, 20090 Milan, Italy; 6 Personalized Medicine, Asthma and Allergy, IRCCS Humanitas Research Hospital, Rozzano, 20089 Milan, Italy; 7 Pediatric Allergy and Clinical Immunology Service, Hospital Sant Joan de Déu, 08950 Barcelona, Spain; 8 Department of Otorhinolaryngology, Head and Neck Surgery, Section of Rhinology and Allergy, Philipps-Universität Marburg, University Hospital Marburg, 35039 Marburg, Germany

**Keywords:** allergen immunotherapy, mechanisms, biomarkers, clinical evaluation, personalized medicine

## Abstract

Allergen-immunotherapy (AIT) is an efficacious and disease-modifying treatment option for IgE-mediated diseases. Among these allergic rhinitis, insect venom allergy, food allergy, and allergic asthma are the most common candidates for AIT. AIT gives rise to clinical immunotolerance which may last for years after the treatment cessation. Mechanisms of AIT include suppression of allergic inflammation in target tissues and stimulation of the production of blocking antibodies, especially IgG4 and IgA. These mechanisms are followed by a reduction of underlying allergen-specific Th2 cell-driven responses to the allergens. Tolerance induction takes place through the desensitization of effector cells and stimulation of regulatory T cells that show their effects by mechanisms involving cell-cell cross-talk, but also other mechanisms, e.g., by the production of immunomodulatory cytokines such as, e.g., IL-10 and TGF-beta. From a personalized medical perspective, there is a need for clinical biomarkers of value in selecting responders and optimizing patient care during AIT. Also, a deeper understanding of underlying mechanistic processes will improve AIT’s future outcomes. In this paper, the current knowledge of mechanisms in AIT is reviewed with a special focus on biomarkers of this therapy.

## 1. Introduction

Allergen-immunotherapy (AIT) is a way to treat IgE-mediated diseases, such as insect venom allergy, food allergy, allergic rhinitis, and allergic asthma, which cause desensitization against allergens. The classical AIT methods are subcutaneous (SCIT) and sublingual allergen immunotherapies (SLIT) [1].

Food immunotherapy is recently approved by FDA (for peanut oral immunotherapy [2]) and it has oral, sublingual, and epicutaneous application routes. Respiratory allergens and Hymenoptera venom allergy were introduced many years ago. For venom immunotherapy (VIT) currently, the only way of application is SCIT but for other forms of AIT, SLIT or SCIT may be preferred [3,4,5]. The primary aim of VIT is to prevent fatal or life-threatening reactions to stings. AIT aims to reduce or abolish allergy signs and symptoms by inducing tolerance. The effectiveness of AIT is not predictable in individual patients. [6].

Blocking IgG4 antibodies exerts their effect by inhibiting IgE-dependent reactions on e.g., mast cells, basophils and B cells. Antigen-specific T-regulatory cells produce interleukin-10 (IL-10) and suppress Th2 immunity, and the immune balance shifts from a Th2-type to a Th1-type immune response [7]. B-regulatory cells are a newly identified cell type and are involved in enhancing immune tolerance by suppressing effector T cells via IL-10, blocking dendritic cell maturation, and producing blocking antibodies. The success of AIT is also related to the reduction of effector cell numbers in target tissues [8].

Although AIT is an effective, safe and disease-modifying treatment, not all patients respond to the therapy significantly. The definition of appropriate biomarkers may help e.g., determine when to discontinue treatment in patients who respond well, predict relapse, and when to apply booster therapy. Currently, no clinical biomarker has been identified and validated to predict clinical response. Candidate biomarkers include e.g., allergen-specific IgE (sIgE), IgE/Total IgE ratio, sIgE/IgG4 ratio, basophil activation tests, some cytokines, serum inhibitory activity for IgE, cellular markers, and provocation tests. However, lack of standardization, reproducibility of the results, the definition of responders and non-responders, and technical difficulties in laboratory methods are the main problems related to the candidate biomarkers [8].

## 2. Mechanisms of AIT

### 2.1. Antibody Responses

**Figure 1:**
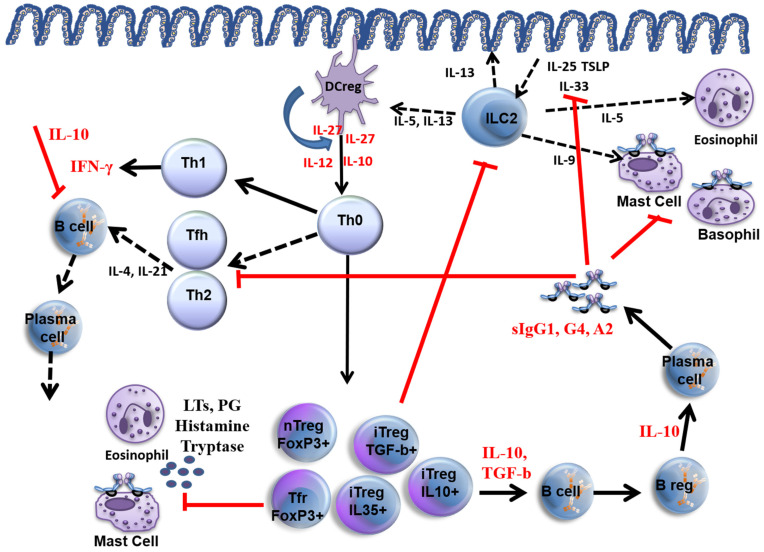
Mechanisms of allergen immunotherapy. Red lines show how allergen immunotherapy inhibits allergic inflammation. Red cytokine levels increase as a result of AIT. Black lines are pathways of allergic inflammation, and black cytokines are those that increase during allergic inflammation. DC reg: Regulatory dendritic cell, ILC2: Innate lymphoid type 2 cell, LTs leukotrienes, PG: prostaglandin, Tfh: T follicular helper cell, nTreg: natural T regulatory cell, iTreg: inducible T regulatory cell, Tfr: follicular T regulatory cell. TSLP: Thymic stromal lymphopoietin.

The main mechanisms of allergen immunotherapy are summarised in Figure 1. During AIT, an early temporary increase in allergen-specific IgE (sIgE) in peripheral blood is observed, followed by a decrease in sIgE over time [9,10]. Allergen-specific IgA, IgG4 antibodies, i.e., blocking antibodies, increase throughout the AIT [11,12,13].

An increase in saliva specific IgA was observed in children undergoing SLIT [14]. Additionally, in a recent study comparing local nasal and systemic IgA1 and IgA2 concentrations after SCIT and SLIT, an increase in IgA was observed in SLIT during and after immunotherapy, but not in the SCIT group [15]. This supports the idea that increased IgA in SLIT may be associated with clinical improvements observed during treatment and may play a role in blocking the binding of allergens to IgE receptors [16].

Blocking antibodies, especially IgG4, block the allergen-sIgE interaction by competing with sIgE. This blockage prevents e.g., cross-linking of allergen-sIgE complexes on basophils and mast cells. As a result, these effector cells’ activation is inhibited. This allergen-specific IgG elevation is seen not only in serum levels but also locally in nasal secretions [17]. Blocking antibodies also prevents the IgE-facilitated allergen presentation to T cells through the FcɣRIIb receptors on B cells. IL-10, a crucial cytokine produced by Tregs and Bregs, is involved in inhibiting allergen-specific effector cells. In addition, IL-10 induces IgG4 production while reducing total IgE and sIgE levels.

In allergen immunotherapy, blocking antibody levels decrease significantly within one year after AIT is discontinued [18,19,20]. However, IgG-related serum IgE inhibitory activity continues for years and is related to clinical response [21]. On the other hand, during venom immunotherapy (VIT), desensitization is related to high IgG4 levels and IgE-Inhibitory activity. When the VIT stops, sIgG4 and IgE-Facilitated allergen binding (IgE-FAB) inhibitory activity return to baseline within months of stopping AIT, and further follow-up showed a more persistent decrease in venom-sIgE levels. This observation suggests an alternative mechanism of prolonged protection other than blocking antibodies [22].

Food-specific IgE levels rise transiently in the early stages of treatment with food AIT, but then decline [23,24,25]. Although low baseline food-specific IgE may be considered a biomarker of tolerance development, lowering food-specific IgE below a certain threshold does not imply the development of tolerance. Food -specific IgG4 levels increase with food AIT [23,24,26]. However, changes in IgG4 levels or the ratio of food sIgE/IgG4 ratio seems not related to the tolerance development. One explanation may be that IgG4 is not associated with tolerance, as it reflects previous allergen exposure and thus reflects less severe allergy at baseline [27].

### 2.2. Cellular Responses

AIT acts by desensitizing effector cells [28], including mast cells and basophils. Not only the number of these cells is influenced, but also the cytokine release thresholds rise over time. At the beginning of rush VIT, basophil numbers start to decrease in the peripheral blood, surface antigens of basophils down-regulate and basophil-derived cytokines IL-13 and IL-4 decrease. Mast cell functions and serum tryptase levels decrease [29,30].

In every form of food immunotherapy i.e., oral immunotherapy (OIT), sublingual immunotherapy (SLIT), and epicutaneous immunotherapy (EPIT) a decrease in basophil activation may be seen [31,32,33]. Studies by peanut OIT and milk OIT/SLIT showed early decreases in basophil activation and this is related to the tolerance development at the end of the studies [33,34]. However, this decreased activation is transient and it rebounds after the cessation of immunotherapy in many studies [28,35,36].

AIT with aeroallergens inhibits both early and late-phase allergic responses at allergic target organs, by suppressing several cytokines and decreasing e.g., mast cell and basophil numbers. This observation suggests that AIT is effective at target organs as well as at the systemic level. AIT with other allergens is expected to have similar mechanisms [8,22,37].

T and B cell responses change during AIT as the immune tolerance develops. A change in immune responses from Th2 to Th1 cell type occurs, which is characterized by an increase in Th1-related interferon-gamma (IFN-γ) and a decrease in Th2-related IL-4 and IL-13 cytokine levels. As Th2 activity reduces, the function and numbers of Treg cell cells increase. Treg cells are critical in peripheral immune tolerance development with distinct subgroups; Natural T regulatory (nTreg) cells, which are transcription factor forkhead box P3 (FOXP3) positive, and inducible T regulatory (iTreg) cells such as IL-10 producing Tr1 cells and TGF-β producing Th3 cells. The major cytokine produced by Tregs is IL-10, which plays an inhibitory role on B cells by blocking the B7/CD28 pathway. This blockage leads to the suppression of dendritic cell maturation, MHC class II expression, and costimulatory ligand activation. Meanwhile, TGF-β suppresses the FcεRI on Langerhans cells, upregulates FOXP3, and RUNX, and helps CTLA-4 expression on T cells.

IL-10 plays a significant role in clinical response and immune tolerance during AIT. IL-10 blockade in peripheral blood reverses the effects of AIT, and allergen-specific proliferative and cytokine responses augment [38]. Following the AIT, CD4+CD25+FOXP3+ Treg cells increase in the inflammation site. This finding supports the idea that Treg cells play an essential role in allergen-specific immune tolerance. All types of allergens used in AIT cause a shift towards a regulatory/suppressor T cell response [8].

In a VIT study, dermal biopsies were taken before and three months after immunotherapy to evaluate allergen-driven alterations in cytokine mRNA expression and cellular differences. The results showed a prominent decrease in IL-4 mRNA expression as well as an increase in IL-10+ cell numbers and a trend of increase for IL-10 mRNA expression [39]. In another study, CD4+CD8+ T lymphocyte changes were analyzed before VIT, at the end of 5 days semi-rush, and at the 6th month of VIT. A significant decrease in IL-4+CD4+ and CD8+ T cell numbers was observed by the end of 5 days of semi-rush VIT. After six months of VIT, an increase in IL-2+IFN-γ+CD4+CD8+ T lymphocytes has been shown, confirming a shift from Th2 to Th1 type immune deviation. IL-10 levels in peripheral blood increased just by the second day of AIT, and by the end of 4th week of treatment, allergen-specific T cells were influenced by the suppressive effect of IL-10 [22].

From the perspective of food IT, one study using a tetramer-based approach found that Ara h 2-specific circulating memory B cells are induced early and transiently in peanut oral immunotherapy and clonal antigen-specific responses to immunotherapy has been shown [40]. In another study, sorting of Ara h 1 or 2 reactive B cells followed by deep sequencing showed that immunotherapy stimulated somatic mutations in IgG4 [41]. These findings support the idea that B cells may play important roles during AIT.

T follicular helper cells (Tfh) are characterized by CXCR5+ surface receptors and they are involved in B-cell maturation and Ig-class switching. A specific type of Tregs, i.e., CXCR5+ FOXP3+ Treg cells, are named follicular regulatory T (Tfr) cells. They move to the germinal centers in the lymph nodes and decrease T and B cell activity. An AIT study showed a significant suppression in Tfh cells following AIT. There is a transformation potential between Tfh and Tfr cells i.e., plasticity and Tfr cells produce more IL-10 compared to Tfh cells. This supports the idea that Tfr cells may have important roles during AIT and immune tolerance development which results in a significant decrease in Th2 responses.

Allergen-specific Breg cells that secrete IL-10 have been identified in beekeepers tolerant to bee stings and patients treated with VIT. Breg cells are CD73-CD25+CD71+ B cells that can suppress allergen-specific CD4+ T cells and stimulate allergen-specific IgG4 production during AIT. Additionally, Bregs produce IL-35 and TGF-beta.

Apart from well-known cell types, there are also some other cells, such as natural killer regulatory cells, which have the capacity for IL-10 production. These cells decrease allergen-induced T cell proliferation through the IL-10 production during AIT, and like other regulatory cell types, they may be involved in tolerance development.

### 2.3. Innate Lymphoid Cells and AIT

Innate lymphoid cell type-2 (ILC2) appears to take part in allergic reactions. The effect of AIT on ILC type 2 was studied in AIT. AIT blocked seasonal increases in ILC2s numbers in effector sites. A decrease in ILC2 number was parallel to the improvement and clinical scores of the patients receiving AIT. Otherwise, ILC1 is the major producer of IFNɣ and TNFα. In fact, the ratio of ILC2/ILC1 is high in perennial AR patients sensitized. This high ratio decreases and turns to normal levels during AIT. This effect is not seen in non-responders to AIT, and they present a similar ILC2/ILC1 ratio to untreated patients. Under the action of retinoic acid, ILC2 cells transform into IL-10-producing regulatory ILCs (ILCregs). These cells can reduce Th2 cells and ILC2 activation. Retinoic acid also stimulates peripheral Treg cell differentiation. By combining these together, ILCregs can participate in tolerance development in AIT mechanisms [8,42]. A recent finding supports this idea. Golebski et al. identified a distinct subset of ILC-2 that produces IL-10 and has regulatory properties that increase after AIT [43].

A distinct innate lymphoid cell type, ILC type 3, may have an important role during immune tolerance development in SLIT. They express CD40L and are located side by side with B cells in the tonsils. ILC3s stimulate IL-15 production in B cells, and IL-15 stimulates the CD40L expression in ILC3s. CD40L+ ILC3s induce IL-10-secreting Breg cells via the CD40L and BAFF-receptor-dependent pathway. ILC3-associated Breg cells are identified by CD27-IgD+IgM+CD24highCD38highCD1d+, and called immature transitional (itBreg) phenotype. This interaction seems to be essential for maintaining immune tolerance for self and harmless antigens and is inappropriate in allergic diseases. ILC3s, Breg cells and Treg cells interact closely in the interfollicular regions of the palatine tonsils. CD40L+ ILC3s may be required in maintaining immune tolerance in the tonsils through the induction of functional itBreg cells. These cells may act not only by cell-to-cell contact via programmed cell death ligand 1, but also by secreting IL-10 for immune tolerance development [42].

### 2.4. Histamine and Histamine Receptors

Desensitization during AIT starts as early as after the first few injections in SCIT. Histamine is an important mediator released from mast cells and exerts its effect through its receptors. Histamine receptor 2 (HR2) stimulation results in desensitization of basophils. Decreased basophil activity parallels clinical scores in AIT. H2R attenuates allergen-specific FcεRI-mediated basophil stimulation. HR2 has important roles in immune tolerance mechanisms. HR2 expression in Th2 cells reduces allergen-induced T cell responses and induces peripheral tolerance by increasing IL-10 production [11,12,13]. Histamine acts via HR2 and increases IL-10 production not only in dendritic cells but also in Th2 cells. It stimulates the suppressive effect of TGF-β on T cells and reduces the production of Th2 cytokines (IL-4 and IL-13 are the main Th2 type cytokines) [8,44].

## 3. Biomarkers for AIT

AIT is an effective method for the treatment of IgE-mediated allergic diseases. However, not all patients respond to the therapy. A validated biomarker is significant from the perspective of personalized medicine, including to obtain an adequate cost/benefit ratio. Surrogate biomarkers may help e.g., to identify good responders, when to stop treatment, predict relapse and when to apply a booster treatment. Currently, there is no defined and validated clinical biomarker to predict clinical response. Proposed biomarkers to assess the efficacy of allergen immunotherapy have been summarized in Table 1.

Despite the existence of several candidate biomarkers, there are challenges regarding standardization, reproducibility of results and identification of responders versus non-responders, and complexity of laboratory methods. Some of these biomarkers are discussed briefly.

Total IgE (tIgE) often increases in allergic patients. During AIT, total IgE levels first increase and then decrease over time. However, its value in diagnosing allergic disease and predicting AIT efficacy is conflicting, and different studies have yielded opposite results [21,45,46,47,48]. Allergen sIgE is the diagnostic method for allergic diseases together with skin prick testing. They also represent the standard tests as an inclusion criterion for AIT. During AIT, sIgE levels increase in the early stages and then decrease gradually throughout treatment. Early rise in sIgE does not associate with the clinical response, and the changes in sIgE levels cannot distinguish responders from non-responders. In the AIT studies, the serum sIgE/tIgE ratio (sIgE/tIgE) associated with rhinitis signs and symptoms scores, and even some cut-off values were defined to predict clinical response. However, conflicting data with other studies bring the need for better-defined studies to use the sIgE/tIgE ratio as a reliable tool [8,22,47,49].

Allergen-specific IgG4 and IgG1 increase during AIT in target organs and peripheral blood [11,12,13]. They have the blocking capacity for effector cells and are involved in developing immune tolerance. However, these antibodies are not considered reliable markers for the AIT response. In some SCIT studies, the increase in sIgG4 was not associated with clinical reactivity. Still, the clinical response was found to be associated with serum inhibitory activity for IgE, even after AIT cessation. This suggests that the concept of the functional significance of sIgG subgroups rather than serum levels is essential for sustained clinical tolerance [50].

A recent systematic review evaluated the clinical utility of microarray B cell epitope mapping as a potential biomarker for food allergy diagnosis, clinical severity, and response to immunotherapy [51]. In one of these studies, peanut oral immunotherapy showed an increase in IgG4 levels, a decrease in IgE level and diversity of Ara h 1, 2, and 3 epitopes at the same time [52]. Additionally, another study evaluated the IgE and IgG4 binding to cows’ milk peptides to assess the responders and non-responders to cows’ milk OIT [53]. In another work, authors offered two sets of IgE binding peptides to predict the ones with the slow response to desensitization and the ones with more adverse reactions during cows’ milk OIT [54].

Inhibition of allergen binding to IgE is evaluated by a validated flow cytometry-based test called Ig-E FAB [55]. It shows IgE-allergen complexes on FcεRII receptors (CD23) in B Cells. IgE-FAB is a promising biomarker, and its effectiveness must be tested in clinical trials. However, it is quite complex and is only available in a limited number of centers [50]. An alternative and simpler method has been developed: the enzyme-linked immunosorbent facilitated antigen binding assay (ELIFAB). In this method, soluble CD23 monomers are attached to a solid surface instead of B cell lines [56]. IgE-BF defines the competition between serum components and sIgE to bind to the allergen and is measured by a solid phase test [57]. IgE-BF increases during AIT and associates with clinical response [58,59]

AIT may prevent basophil activation through the allergen sIgG antibodies. Basophil activation may be detected either by histamine release or by identifying basophil surface markers CD63 and CD203c by flow cytometry assays [60,61,62]. A new flow cytometric method called the diamine oxidase (DAO) test is another alternative to see basophil activity during AIT. DAO uses histamine as its substrate and binds to intracellular histamine. Basophil activation leads to histamine release from cells [63,64] with a change in intracellular DAO levels indicating the activation level of the basophils. There is inconsistency in results between studies concerning basophil activation. At least in part, the conflicting results may be associated with the type of AIT. SCIT studies give more promising results compared to SLIT, as SLIT appears less effective in basophil suppression.

A shift from Th2 inflammation to Th1 inflammation occurs during allergen immunotherapy. During this transition, cytokines and chemokines play an important role. Not only Th1-related cytokines (e.g., IFN-ɣ, IL-12) [65,66], but also Treg-related cytokines (e.g., IL-10 and TGF-β) are increased [67,68]. There is also a decrease in Th2 response-related cytokines (e.g., IL-4, IL-13, IL-9, IL-17, TNF-α) [66]. However, there is inconsistency between studies and some of them do not find a strong relationship between clinical response and expected cytokine profile [69,70].

Chemokines are small proteins that have the ability to induce the migration of cells (chemoattractive functions) that play an important role in the pathophysiology of allergic disorders. Chemokines may increase (eotaxin [71], complement C4a [72], leptin [73], thymus and activation regulated protein (TARC) [71], transthyretin [72]); decrease (complement C3a, C5a [74] eotaxin [75]) or may be unchanged (adiponectin [76], tryptase [75], eosinophilic cationic protein (ECP) [75], soluble HLA molecules [77]) in different studies during AIT. Currently, there is no confirmed consensus on potential cytokine, chemokine or molecule biomarkers to predict clinical response to AIT.

Candidate cellular biomarkers related to the AIT response include regulatory T cells (Tregs), regulatory B cells (Bregs), native lymphoid cells (ILCs), dendritic cells, T helper cells (Th1, Th2), T follicular helper (Tfh) and T follicular regulator (Tfr) cells [7,8].

Breg cells appear to be important for the development of allergen tolerance and their function is mainly associated with the following possible mechanisms: IL-10-mediated suppression of effector T cells (including Th2 responses), stimulation of Treg cells, IL-10-mediated blockade of dendritic cells maturation, modulation of Tfh responses, and anti-inflammatory production of IgG4 antibodies [78].

All of these cells are modified during AIT. Some changes in these cells have been reported to distinguish between treatment groups and are generally associated with treatment outcomes. However, there is insufficient data to correlate any of these cells with clinical efficacy, and they are technically challenging to identify. They are currently unlikely to represent biomarkers.

Some markers related to polarized dendritic cells have been investigated to test the efficacy of SLIT as well. Component 1 (C1Q), receptor stabilin1 and STAB1 were found to be elevated in the blood samples of clinically responding patients, contrary to non-responding or control group patients. However, these findings need to be validated in further studies and practice [8,79].

In-vivo biomarkers evaluate the response to AIT directly in the involved organ by provocation with the relevant allergen. They include skin prick tests (SPT), intradermal tests, and conjunctival, nasal and bronchial provocation tests. In general, these tests are used in clinics for diagnostic purposes. Additionally, in-vivo biomarkers may also be used for the evaluation of the clinical efficacy of AIT. There are, however, several protocols that have been published on different challenge models. Therefore, there is a need for standardization and validation of these different provocation models.

Recently, the use of environmental challenge chambers (ECC) has become an alternative method to expose the patient to a predefined allergy dose for evaluation of provocation tests. An ECC represents a provocation test that may closely simulate natural exposure. An EEC may be used to expose the patient to a stable and reproducible allergen amount under the same environmental conditions. Additionally, as seen in some studies, ECC may determine the starting time for AIT studies. Comparable clinical scores were achieved in a study conducted by ECC with an allergen dose similar to environmental exposure. For AIT, further validation of the treatment effect size as assessed in the EEC challenges is needed to relate it to effect sizes found under natural exposure in field trials [80,81,82].

There is a clear need to find reliable biomarkers for food AIT to determine which patients will respond better and to see the efficacy of immunotherapy after treatment is discontinued. Currently, the standard approach to measuring efficacy is via pre-treatment and post-treatment double-blind, placebo-controlled food challenges. This approach can sometimes be time-consuming, resource-intensive, and carries the risk of serious allergic reactions, including anaphylaxis [79].

From a VIT perspective, sting challenges are a reliable method and gold standard for determining the efficacy of VIT [4,83]. In addition, field stings can provide information about the effectiveness of the treatment. Early sting challenges are fairly reliable for determining the efficacy of VIT [84], but repeated sting challenge trials three to five years after treatment can poorly identify patients with relapse [85,86]. Sting challenges are important not only for demonstrating treatment efficacy but also for health-related quality of life, and a passed challenge improves patients’ confidence as well [87].

Recently, epigenetic studies and omics technology also offer some new potential biomarkers for AIT. A dual SLIT study with grass and house dust mite allergens resulted in the induction of memory Treg cells via reduced DNA methylation of the CpG regions of the FOXP3+ locus [88]. A recent study of SCIT measured serum sample metabolites from patients who did and did not respond well to AIT and found that L-Tyrosine was an indicator of response and decreased in those who responded well. In addition, AIT was found to be associated with NO and nitric oxide synthase metabolism [89]. Eicosanoids, such as prostaglandins and leukotrienes, are important molecules for innate immune responses, and especially 12(S)-hydroxyeicosatetraenoic acid (HETE) and 15(S)-HETE is reduced after treatment with SCIT and they may be promising candidate molecules [90].

**Table 1:** Biomarkers to assess the efficacy of allergen immunotherapy. (Adapted and modified from [7,91]).

Possible Biomarkers and Examples	Advantage	Limitation	References
**1.** **IgE** **Total IgE** **Specific IgE** **sIgE/tIgE**	-Easy test to run, serum-based laboratory test -sIgE/tIgE seems to be reliable and promising marker to show clinical response	-sIgE rising at the beginning of AIT and not associated with the clinical response -sIgE/tIgE not validated yet. -Need for equivalence studies between tIgE units and sIgE units	[12,21,45,46,48,49,92,93,94,95,96]
**2.** **IgG and subclasses** **sIgG1** **sIgG4** **sIgE/sIgG4**	-Easy test to run, serum-based laboratory test -sIgG4 informative for allergen exposure -Currently, there is a commercial kit for sIgG4	-It is not clear whether the sIgG4 levels and symptom and medication scores associate well. Need for further studies -Insufficient data on other IgG subsets -Limited data on local antibody levels -Low sIgG4 levels is a difficulty for measurement	[12,13,18,19,20,21,47,48,60,68,97,98,99,100]
**3.** **Serum Inhibitor activity** **IgE-FAB** **IgE-BF** **ELIFAB**	-Serum-based laboratory test -High reproducibility is an advantage for IgE-FAB -IgE-FAB and IgE-BF associated with the clinical responses and reported in previous studies	-IgE-BF is not commercially available -It is not clear if IgE-FAB discriminates good responders -Despite the association between clinic response and IgE-FAB the number of studies is limited	[11,12,13,16,21,55,56,57,59,60,61,101,102,103,104]
**4.** **Basophil activation tests** **CD63** **CD203c** **CD107** **Diamine Oxidase**	-Shows basophil activation with FcεRI mediated in-vivo response -Small amount of blood needed	-Test variability between different centers -Difficult technique -Lack of dose-response curves -Unresponsive basophils in some people	[11,60,63,64,105,106,107,108,109,110,111,112]
**5.** **Cytokines, chemokines and molecules** **ECP, Eotaxin** **IL-2R/IL-2** **IL-4, IL-5, IL-9, IL-13, IL-17** **IFN-** **γ** **, IL-12** **TGF-** **β** **, IL-10** **CCR3** **TARC** **Tryptase**	-Useful to understand the mechanisms of AIT -Local cytokine production may relate more to clinical manifestations	-There is no cytokine, chemokine or molecules identified to predict clinical response until now -Results may differ between centers and studies. -Specific T cell originated cytokine levels may be very low to be determined	[71,72,73,74,76,113]
**6.** **Cellular biomarkers** **Tregs** **B regs** **Dendritic cells** **ILCs**	-Tregs are very important in tolerance development -Bregs take very important roles in the mechanism of AIT -Treg and Breg cells may be more useful for drug development	-Technical difficulties -No data to show the association between Tregs and clinical response -Tregs appear very early in AIT so difficult to be a biomarker -Very low frequency of Tregs and Bregs	[7,78,79]
**7.** **In vivo biomarkers** **SPT** **Intradermal tests** **Conjunctival provocation tests** **Nasal Provocation tests** **Bronchial provocation tests** **Environmental Challenge Chambers (ECC)** **Food challenges** **Sting Challenges** **Allergen Challenges**	-Standardised environmental factors -Seasonal pollen variations may be avoided -Surrogate markers of clinical response to AIT -ECC decrease variability in clinical studies, and they allow dose-response studies. -Food challenges help to find threshold levels for safe consumption -Early sting challenges are fairly reliable	-Mimics natural exposure but not the same -Lack of standardization for some allergen challenges -Cost of ECC is high, some reproducibility problems exist -Food challenges sometimes may be time-consuming, resource-intensive, and carries the risk of serious allergic reactions, including anaphylaxis -As time passes after treatment cessation sting challenges are poor to demonstrate relapse	[4,79,81,82,83,85,86,87,88,89,90]

AIT: Allergen immunotherapy, CCR3: C-C chemokine receptor type 3, ECP: Eosinophilic cationic protein, ELIFAB: Enzyme-linked immunosorbent-facilitated antigen-binding assay, DAO: Diamine oxidase, IgE-BF: IgE-blocking factor, FAB: facilitated antigen binding, IFN-γ: Interferon-gamma, ILCs: Innate lymphoid cells, SPT: Skin prick tests, TARC: thymus and activation regulated protein, TGF-β: Transforming growth factor-beta.

## 4. Conclusions

As the only disease-modifying treatment option for individuals with IgE-mediated allergy, allergen immunotherapy has been shown to be both clinically effective and safe. In general, AIT is an effective treatment option. However, some patients do not respond to AIT well. There is an increasing understanding of underlying mechanisms in AIT laying the path for biomarkers for indication, monitoring, and optimizing the course of treatment. Through the modulation of innate and adaptive immune responses. To date, there has been no clear relationship between the immunological changes observed and responders and non-responders to AIT. However, IgE-FAB represents a promising biomarker. Antibody and cellular responses, molecules enable us many insights into the mechanisms of AIT but cannot be applied as biomarkers in a clinical setting yet. Finally, the use of the challenge test is still limited.

Further research is needed to confirm and interpret the possible association with biomarkers and clinical response to AIT.

## Data Availability

Not applicable.
